# An Artificial Intelligence-Based Prognostic Model for Prediction of Functional Glaucoma Progression From Clinical and Structural Data

**DOI:** 10.1016/j.ajo.2025.12.026

**Published:** 2026-01-01

**Authors:** VAHID MOHAMMADZADEH, SEAN WU, SAJAD BESHARATI, MAHSHAD RAFIEE, YASAMIN BANAEI, ARTHUR MARTINYAN, JANE ZOU, EVELYN KUNG, KIUMARS EDALATI, ESTEBAN MORALES, FABIEN SCALZO, JOSEPH CAPRIOLI, KOUROS NOURI-MAHDAVI

**Affiliations:** Glaucoma Division (V.M., S.B., M.R., Y.B., A.M., J.Z., E.K., K.E., E.M., J.C., K.N.M.), Stein Eye Institute, David Geffen School of Medicine, University of California Los Angeles, Los Angeles, California, USA; Department of Computer Science (S.W., F.S.), Pepperdine University, Malibu, California, USA; Department of Computer Science (F.S.), University of California Los Angeles, Los Angeles, California, USA; Department of Ophthalmology and Vision Sciences (V.M.), University of Louisville, Louisville, Kentucky, USA

## Abstract

**PURPOSE:**

Integration of various sources of information for prediction of disease progression is an unmet need in glaucoma diagnostics. We designed a deep learning-based prognostic model incorporating clinical and structural data for forecasting functional glaucoma progression and compared its performance to clinicians.

**DESIGN:**

Retrospective, comparative cohort study of prognostic accuracy.

**SUBJECTS:**

We included 1599 eyes (908 patients) with definite or suspected glaucoma with ≥5 24-2 visual fields (VF) and 3 or more years of follow-up.

**METHODS:**

VF mean deviation (MD) rates of change were estimated with linear regression. Sequential MD rates of change were estimated with each series spanning only 5 years of follow-up. VF progression was declared when four sequential statistically significant negative MD slopes were observed, and slope for the entire follow-up was significant. A convolutional neural network pretrained on ImageNet was designed to predict VF progression using baseline clinical and demographic data, disc photographs, and optical coherence tomography-derived global and sectoral retinal nerve fiber layer and macular thickness measurements. In addition, average intraocular pressure and treatment information during follow-up were put into the model. The same data for a subset of patients was provided to two clinicians to independently predict future progression. The model was validated on a separate cohort of eyes in which optical coherence tomography imaging was done with a different device (291 eyes).

**MAIN OUTCOME MEASURES:**

Model’s area under receiver operating characteristic curves (AUC), accuracy, and area under the precision and recall curves.

**RESULTS:**

Average (SD) baseline MD and number of VF exams were −3.5 (4.9) dB and 10.1 (4.7). 399 eyes (25%) deteriorated. The best-performing model incorporated baseline disc photographs, and retinal nerve fiber layer and macular thickness: AUC, 0.839 (0.7710-0.906), accuracy, 76.0% (62.0%-85.0%), and area under the precision and recall curves, 0.558 (0.385-0.733). Deep learning model significantly outperformed clinical graders (AUC : 0.629 [0.531-0738], *P* < .001 and 0.680 [0.584-0.776], *P* = .001, for grader one and two, respectively). Model performance was similar on the validation cohort (AUC: 0.754 [0.671-0.837], and accuracy: 77% [71%-82%], respectively, *P* = .122). The model performed well when predicting fast-progression, defined as MD rate <−1.0 dB/y (AUC: 0.869 [0.792-0.947]).

**CONCLUSIONS:**

Our newly designed deep learning model can combine baseline demographic and clinical data with widely available structural measurements and provide clinically relevant information for the prediction of glaucoma progression.

## INTRODUCTION

Timely detection of glaucoma progression remains a crucial clinical endeavor in the care of glaucoma patients; it would allow clinicians to adjust patient monitoring frequency and adjust treatment accordingly. This task is all the more challenging in the advanced stages due to the diminished efficiency of functional and structural measurements.^1,2^ Visual field (VF) testing remains the primary tool for tracking functional glaucoma progression; however, measurement variability significantly increases with worsening disease stage complicating the task of identifying disease deterioration.^3–5^ To date, clinical and demographic data and functional and structural measurements have been used in various combinations to predict glaucoma progression.^6–8^

In the Ocular Hypertension Treatment Study, older age, vertical and horizontal cup-disc ratio, greater pattern SD (PSD), thinner central corneal thickness (CCT), and higher intraocular pressure (IOP) predicted subsequent development of primary open-angle glaucoma (POAG).^9,10^ A subsequent study identified additional baseline factors that increased the risk of future progression; these included presence of exfoliation syndrome, bilateral disease, and worse perimetric mean deviation (MD) at baseline; notably, frequent disc hemorrhages observed during follow-up also elevated the risk.^11,12^ A later study emphasized the significance of peak IOP, thinner CCT, disc hemorrhages, and beta-zone parapapillary atrophy as predictors of disease deterioration highlighting the complex interplay of factors influencing the glaucoma trajectory.^13^ Technological limitations have to date precluded the incorporation of imaging data into functional prognostic studies. Recently, a polygenic risk score has been introduced as a significant predictor of higher POAG risk in patients with ocular hypertension, potentially paving the way for improved forecasting of the onset of glaucoma.^14, 15^

Optic disc photographs (ODP) have been traditionally used for recording and assessing glaucomatous structural changes over time.^16–18^ Optic disc photos are widely available and are not dependent on sophisticated algorithms for interpretation. However, there is a high interobserver variability for detecting serial glaucomatous change with the ODPs.^19–24^ Optical coherence tomography (OCT) imaging of the retinal nerve fiber layer (RNFL) is now widely used for monitoring structural changes in glaucoma.^25–31^ Recent studies after widespread availability of OCT devices have confirmed that loss of macular retinal ganglion cells frequently starts early in the course of glaucoma; hence macular OCT imaging is commonly used for both detection of glaucoma and monitoring disease progression.^32–38^ There is an interest in combining various available structural modalities for detecting or forecasting glaucoma progression.^39–41^ We found that combining rates of RNFL and macular thickness change led to a higher performance for detecting VF progression when compared to rates of change of either modality separately.^40^

Deep learning (DL) models have enhanced our capability to analyze ophthalmic images with greater precision for a range of clinical objectives.^39, 42^ Some recent studies used artificial intelligence (AI) models, more specifically DL models, for enhancing detection and prediction of glaucoma deterioration.^43–50^ We demonstrated that prognostic models integrating baseline and longitudinal structural and functional data with DL models could predict glaucoma progression with clinically relevant performance.^41,50^ No previous study has explored the integration of imaging data with clinical and demographic information for this purpose. Our study aims to advance and refine our prediction algorithm by incorporating baseline clinical and demographic data along with various structural measurements to enhance prediction of future functional glaucoma progression.

## METHODS

One thousand five hundred and ninety-nine eyes (from 908 patients) from Stein Eye Institute’s clinical database were included. The study was approved by the University of California Los Angeles’s Institutional Board Review and adhered to the Declaration of Helsinki and the Health Insurance Portability and Accountability Act policies. The study data were acquired from January 2010 through January 2020, and data collection was carried out in 2021. The study was started in February 2023 and finalized in October 2023. The findings are reported according to Strengthening the Reporting of Observational Studies in Epidemiology (STROBE) and transparent reporting of a multivariable prediction model for individual prognosis or diagnosis-AI (TRIPOD + AI) statement checklist (Supplementary Table 1 Material).

Study patients were required to have a minimum of five VF visits with at least 3 years of follow-up. All eligible eyes had an ODP, an RNFL, and macular OCT images at baseline. VFs were not reviewed by clinicians due to the very large volume of data. VFs with false positive rates > 15% were excluded from the study. Only participants with a diagnosis of POAG, normal tension glaucoma, pigmentary glaucoma, pseudoexfoliative glaucoma, or primary angle closure glaucoma, based on ICD-9 and 10 codes, were eligible for the study.

### STRUCTURAL DATA:

#### ODPs:

ODPs were acquired with two devices, the Zeiss 450 Fundus Camera and the Zeiss FF 450plus Fundus Camera with VISUPAC Digital Imaging System (both from Carl Zeiss Meditec, Dublin, CA). Photographs that were acquired on film before 2013 were previously digitized. Image quality was reviewed, and low-quality images were excluded. Thirty-three ODPs (1.5%) were excluded from the initial dataset; 24 out of those 33 were acquired within the last 5 years of the study. The field of view for the optic disc images varied between 20° and 30°. All the digital ODP were input into the DL model at 256 × 256-pixel resolution.

#### RNFL OCT imaging:

Circumpapillary RNFL (cp-RNFL) OCT imaging was carried out with Cirrus high-definition OCT (Carl Zeiss Meditec, Dublin, CA). The Optic Disc Cube 200 × 200 algorithm scans the optic disc and peripapillary retina; the volume scan is centered on the optic disc. The volume scan consists of 200 linear horizontal B-scans, each containing 200 A-scans; the scanned region is 6 × 6-mm in size in an emmetropic eye. Cirrus OCT’s proprietary software automatically segments the cp-RNFL, delineating the area between the internal limiting membrane and the outer boundary of the RNFL. Scans with a signal strength of less than 6 were excluded. Circumpapillary RNFL measurements at 12-clock-hour sectors and the global cp-RNFL thickness were used in this study.

#### Macular OCT imaging:

Cirrus HD-OCT’s Macular Cube 200 × 200 algorithm consists of 40,000 A-scans in a 6 × 6 × 2 mm cube centered on the fovea. A-scan data were subsequently segmented by Carl Zeiss Meditec’s proprietary software, which delineates the area between the inner boundary of the ganglion cell layer and the outer boundary of the inner plexiform layer (GCIPL). The Ganglion Cell Analysis algorithm reports the GCIPL layer thickness within six wedge-shaped sectors in a 4.8 × 4.0 mm ellipse, excluding an inner elliptical annulus 1.2 × 1.0 mm in diameter. In addition to the sectoral measurements, global and minimum GCIPL thickness measurements were used as input for the DL algorithm. OCT images were not reviewed clinically due to the large number of scans and to make the study findings as similar to the real-world scenario. For Cirrus’ RNFL and macular OCT images, we included scans with signal strength ≥7.

### CLINICAL AND DEMOGRAPHIC DATA:

Clinical and demographic data relevant to subsequent glaucoma progression were manually extracted from the electronic medical records for the baseline visit. These included age, gender, race, IOP, CCT, VF, MD, and PSD. Gender refers to a multidimensional construct encompassing an individual’s selfidentified gender identity and the socially defined roles, behaviors, and expressions associated with that identity To account for the influence of treatment on disease course, both baseline treatment and treatment modifications during follow-up were incorporated into the model. Baseline treatment was defined as being on IOP-lowering medication at the baseline visit or having undergone glaucoma laser or surgical intervention in the 3 months preceding the baseline visit. Treatment during follow-up included any escalation in medical therapy (initiation of drops or increase in the number of medications) as well as any laser or surgical intervention performed after baseline. These treatment variables, along with average IOP during follow-up, were included as model inputs to ensure that therapeutic changes were appropriately considered in the prediction framework and to address the bias introduced by “confounding by indication.”

### DEFINITION OF GLAUCOMA PROGRESSION:

We defined functional glaucoma progression based on rates of change of VF MD with univariable linear regression of MD against time. In order to maintain the linearity of MD rates, consecutive MD slopes were calculated such that each slope included only a 5-year interval (eg, [0-5 years], [1-6 years], [2-7 years], etc.). An eye was classified as progressing if it demonstrated four consecutive, statistically significant negative MD slopes and also showed a statistically significant negative slope over the entire follow-up period. For patients who had both eyes enrolled in the study, both eyes were required to be either stable or progressing; otherwise, that particular individual was excluded from all analyses. The same approach was repeated for defining fast progression, this time defining statistically significant MD rates as MD slope less than −1.0 dB/y. For the fast progressor dataset, the stable group included eyes that were defined as not having statistically significant negative MD rates and eyes that had statistically significant negative rates between 0 and −1 dB/y.

### THE DL MODEL:

#### DL model input—

We considered different sets of inputs for training the model. The first sets of model input combined demographic and clinical data with either ODP, RNFL OCT, or macular OCT measurements. We then utilized demographic/clinical data with various combinations of ODP and RNFL and macular OCT measurements as input for the DL models. The most complex input for training the DL model consisted of the combination of demographic/clinical data with all the available structural data. Patients were split into 80%/20% bins for training and testing datasets. The same split was used for all the models.

**FIGURE 1.**
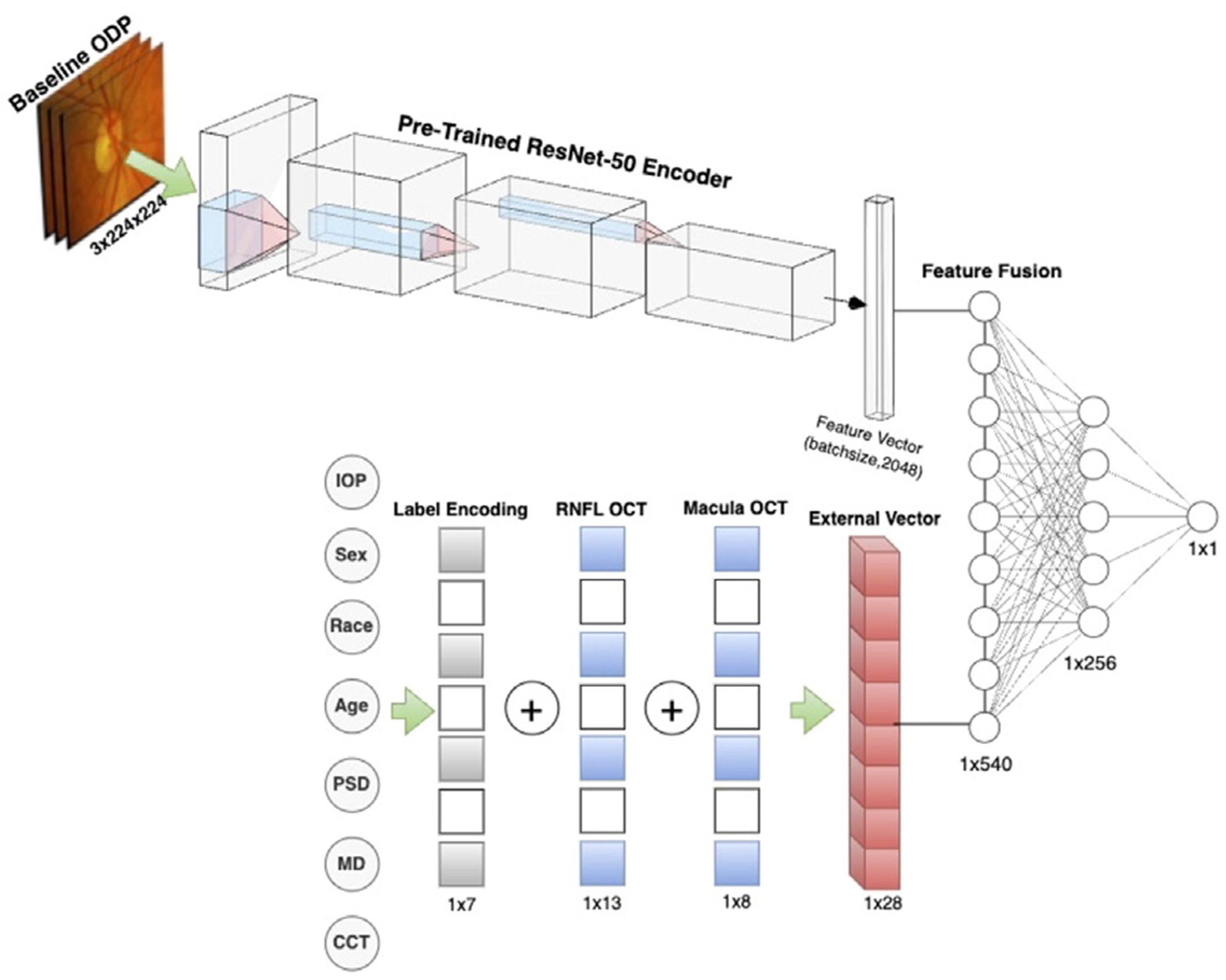
The structure of the deep learning model, including transfer learning for evaluating optic disc photos, a Siamese Neural Network for incorporating demographic data, retinal nerve fiber, and macular optical coherence tomography, and a final convolutional neural network for accommodating the output from the previous networks.

Our DL model leverages transfer learning from the residual neural network family and utilizes feature fusion to capture multiple data modalities, including ODPs, OCT measurements, and demographic/clinical data. More specifically, we used ResNet-50,^51^ which has been proven to handle ODPs well during training. One particular reason we chose the convolutional neural network rather than the transformer architecture is that spatial hierarchies in the ODPs are crucial for glaucoma prediction, so it would benefit from the inductive biases of ResNet-50’s convolutional layers.^52^ For this task, we froze the pretrained convolutional weights because they were trained on ImageNet.^53^ Freezing the weights allows us to better leverage the feature extraction abilities of the network by adapting only the dense head of the network. We then trained the fully connected layer of the network for progression prediction. The first step of the model is encoding binary or categorical demographic information, such as sex and race, into numerical values. Next, we fused the age, MD, demographic, and OCT data together in a single numerical vector (28 features). We edited the final layer of ResNet-50 to output 512 features and combined those 512 features with the numerical vector. The last step was to pass these concatenated features through a feed-forward neural network. The architecture converged from 540 to 512 features, 512 to 256, and finally from 256 features to one node, which represented glaucoma progression (binary yes or no). We included a rectified linear unit activation function between each feedforward layer (Figure 1).

We trained the multimodal neural network using a binary cross-entropy loss with logits, which incorporates an internal sigmoid activation function. To address the class im-balance between progressing and nonprogressing eyes (with only 9.8% of cases exhibiting fast progression), we leveraged a cost-sensitive approach by setting the pos-weight parameter in the BCEWithLogitsLoss function. This parameter increases the penalty associated with misclassifying progressing eyes, which encourages the model to give greater attention to these minority cases. Given the higher clinical importance of correctly identifying fast progressors, this weighting scheme appropriately biases the model toward sensitivity over precision. Model optimization was performed using a learning rate scheduler that reduced the learning rate upon plateauing of the validation area under receiver operating characteristic curves (AUC), enabling more stable convergence during training.

#### Model performance—

We estimated the AUC for assessing the performance of prediction models for the test dataset, which plots the true positive rate (sensitivity) against the false positive rate (1—specificity). In addition, sensitivity, specificity, and accuracy were calculated from the confusion matrices with a threshold of 0.5 for all progressors and 0.71 for the models predicting fast progression. We also report the partial AUC, which is the area under the receiver operating characteristic curve where the specificity is higher than 90%. In order to address inflation of type 1 error from intereye correlation while comparing AUC between models, we used Obuchowski’s method, which is a statistical test for correlated ROC curves in clustered data.^54^ We calculated 95% CIs (CI) by bootstrapping measurements 5000 times. We further calculated the precision (positive predictive value), recall (sensitivity), and the area under the precision-recall curve (AU-PRC) for the best-performing model (demographic/clinical data + ODP + RNFL OCT + macular OCT) predicting any progression (MD rates <0 dB/y) and the one forecasting fast progression (MD rates <−1 dB/y).

#### Comparison models—

We designed a logistic regression model and incorporated all the baseline demographic and clinical data, VF, MD, and PSD, along with baseline RNFL and macular thickness measurements as input. Additionally, we used a lasso logistic regression model to evaluate whether baseline structural, functional, and clinical parameters were associated with disease progression. Lasso regression was selected because it is well-suited to situations with many potentially correlated predictors; by applying a penalty to the regression coefficients, it automatically reduces the weight of less informative variables and can shrink some coefficients entirely to zero, effectively performing variable selection. This helps avoid overfitting and increases the interpretability of the model. The AUC of these models was compared with the AUC of the best-performing AI model with Obuchowski’s method.

#### Clinical classification—

ODPs, global and sectoral RNFL and macular OCT thickness measurements, baseline MD and PSD, and clinical and demographic information, including surgical or laser treatments during follow-up (0/1 variable), an increase in medical treatment (0/1 variable), and average IOP during treatment were provided to two glaucoma specialists (KNM and JC). Each specialist independently determined whether an eye would remain stable or show progression over time. The AUC of their classifications was compared with that of the DL model using the same data. Interobserver agreement between the two specialists was assessed using Cohen’s kappa.

#### Testing on a separate dataset with a different OCT device—

We created a separate cohort of 248 glaucoma eyes (158 patients) meeting the same inclusion criteria, except that the RNFL and macular OCT volume scans were acquired with Spectralis OCT (Heidelberg Engineering). There was no overlap between the 158 patients in the validation subset and the 908 individuals in the original cohort. RNFL volume scan data were derived from a single circular peripapillary B-scan 12° in diameter and consisted of 768 A-scans. To make the RNFL OCT thickness measurements consistent with those of the Cirrus HD-OCT, we divided the 768-pixel measurements into 12 clock-hour sectors and estimated the global RNFL thickness by averaging all the A-scan measurements. Any RNFL scan with a quality score <15 was excluded. The Posterior Pole Algorithm of the Spectralis SD-OCT acquires 61 horizontal B-scans spanning a 30 ° × 25° wide area, parallel to the fovea-Bruch’s membrane opening axis; each B-scan consists of 768 A-scans. The B-scans are repeated 9 to 11 times to decrease speckle noise and improve the image quality. Good quality macular Spectralis OCT images were defined as those with a quality factor >15. Segmentation of the individual retinal layers was carried out with the Glaucoma Module Premium Edition software; thickness data were averaged, and an 8 × 8 grid of thickness measurement was created for the layer of interest, providing 64 3° × 3° super-pixels in the central 24 × 24° of the macula. Data was exported as XML files, and the right-eye format was used for all eyes. We added the GCL and IPL thickness measurements to achieve GCIPL thickness measurements. To make the Spectralis data as similar to the display of Cirrus HD-OCT as possible, we selected the central 6 × 6 superpixels and averaged the values to estimate global and minimum GCIPL thickness, along with GCIPL thickness in the superior and inferior hemiretina, and superior temporal, superior nasal, inferior temporal, and inferior nasal sectors. We tested all the various DL models on this dataset and calculated the same performance metrics.

A *P* value less than .05 was considered statistically significant. Our analyses were exploratory; additionally, we relied on the magnitude of the AUC for comparison of various models in addition to the *P* values found on such exploratory analyses. Therefore, we did not correct for multiple comparisons.

## RESULTS

One thousand five hundred and ninety-nine eyes (from 908 patients) were included in this study. Table 1 provides the clinical and demographic characteristics of the study patients. Based on the progression criteria, 25.0% (399 eyes) demonstrated deterioration of glaucoma. Average (SD) follow-up time and baseline VF MD for stable and progressing eyes were 9.7 (4.6) and 11.4 (4.7) years (*P* < .001) and −3.3 (4.9) and −4.1 (4.9) dB (*P* < .001), respectively. The mean (SD) global RNFL and macular thickness measurements for the stable and progressing eyes were 79.0 (14.9) and 70.0 (13.5) μm (*P* < .001) and 68.2 (12.9) and 61.8 (12.8) μm (*P* < .001), respectively. One hundred fifty-seven eyes (9.8%) were identified as fast progressors. The average (SD) baseline MD and age in this subset were −5.5 (5.0) dB and 60.2 (9.6) years, respectively.

**FIGURE 2.**
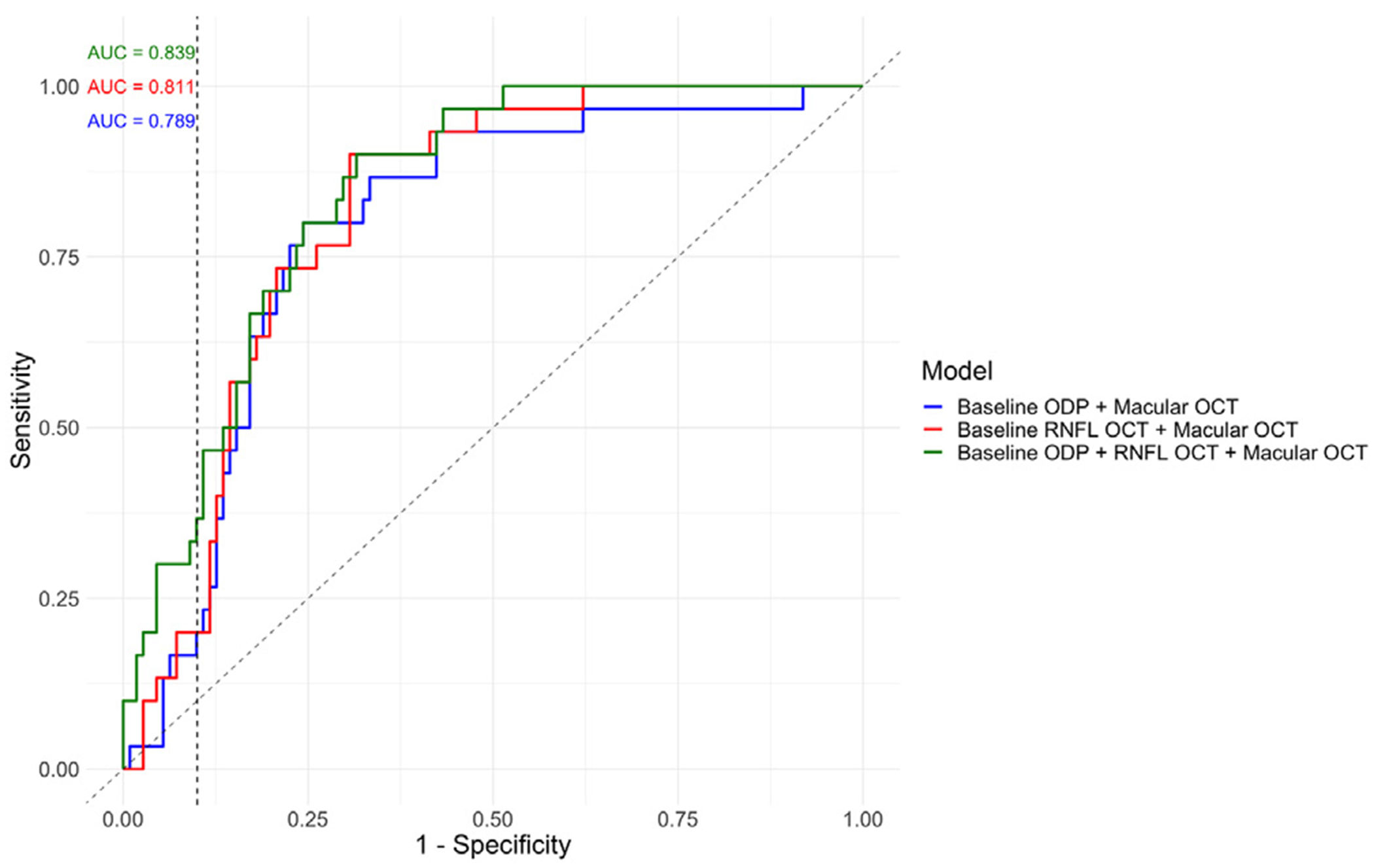
The receiver operating characteristics curves for the top three performing deep learning models for prediction of future glaucoma progression. The portion of the ROC curve to the left of the vertical line demonstrates the partial ROC curves, where specificity is higher than 90%.

Table 2 displays the performance of different DL models for predicting glaucoma progression. The highest performing model was the one incorporating all three structural data along with the demographic/clinical information. The AUC (95% CI) for this model was 0.839 (0.771-0.906) and was significantly higher than the models that utilized only ODP or RNFL OCT modalities (*P* = .003 and 0.001, respectively). The performance of the best performing (AI) model was significantly higher than the logistic regression model (AUC = 0.635 [0.447-0.821], *P* = .049) and Lasso regression model (AUC = 0.732 [0.630-0.834], *P* = .006). This DL model also demonstrated significantly higher performance than the clinical classification by two glaucoma specialists (AUC = 0.629 [0.531-0738], *P* < .001, and 0.680 [0.584-0.776], *P* = .001, respectively). The Cohen Kappa agreement (95% CI) between the graders was 0.28 (0.12-0.44). The sensitivity, specificity, and accuracy of the best-performing model were 90.0% (73.0%-100.0%), 71.0% (53.0%-86.0%), and 76.0% (62.0%-85.0%), respectively. The AI model incorporating only demographic data had an AUC of 0.657 (0.560-0.754), which was significantly worse than the best-performing model (*P* < .001). Models with only one structural modality, along with the demographic/clinical information, demonstrated lower AUCs (0.664 [0.550-0.777], 0.732 [0.632-0.831], and 0.781 [0.690-0.871] for ODP, RNFL OCT, and macular OCT, respectively). Combining any pair of structural data resulted in a higher performance compared to individual-structure models (Table 2). Figure 2 displays the receiver operating characteristic curves (ROC) for the top 3 DL models. The portion of the ROC curve to the left of the vertical line demonstrates the partial ROC curves, ie, the ROC region where specificity is higher than 90%. The partial AUC (pAUC, [95% CI]) for the most complex model utilizing all three structural modalities was 0.023 (0.010-0.042) (Supplementary Table 2).

Table 3 provides the performance metrics for all DL models predicting glaucoma progression in the fast-progressing eyes. The AUC and accuracy (95% CI) of the model that included all three structural modalities along with the demographic/clinical data were 0.869 (0.792-0.947) and 77.0% (69.0%-83.0%), respectively, and its performance was higher than other less complex models. The pAUC for all the models predicting fast progression is presented in the Supplementary Table 2.

**FIGURE 3.**
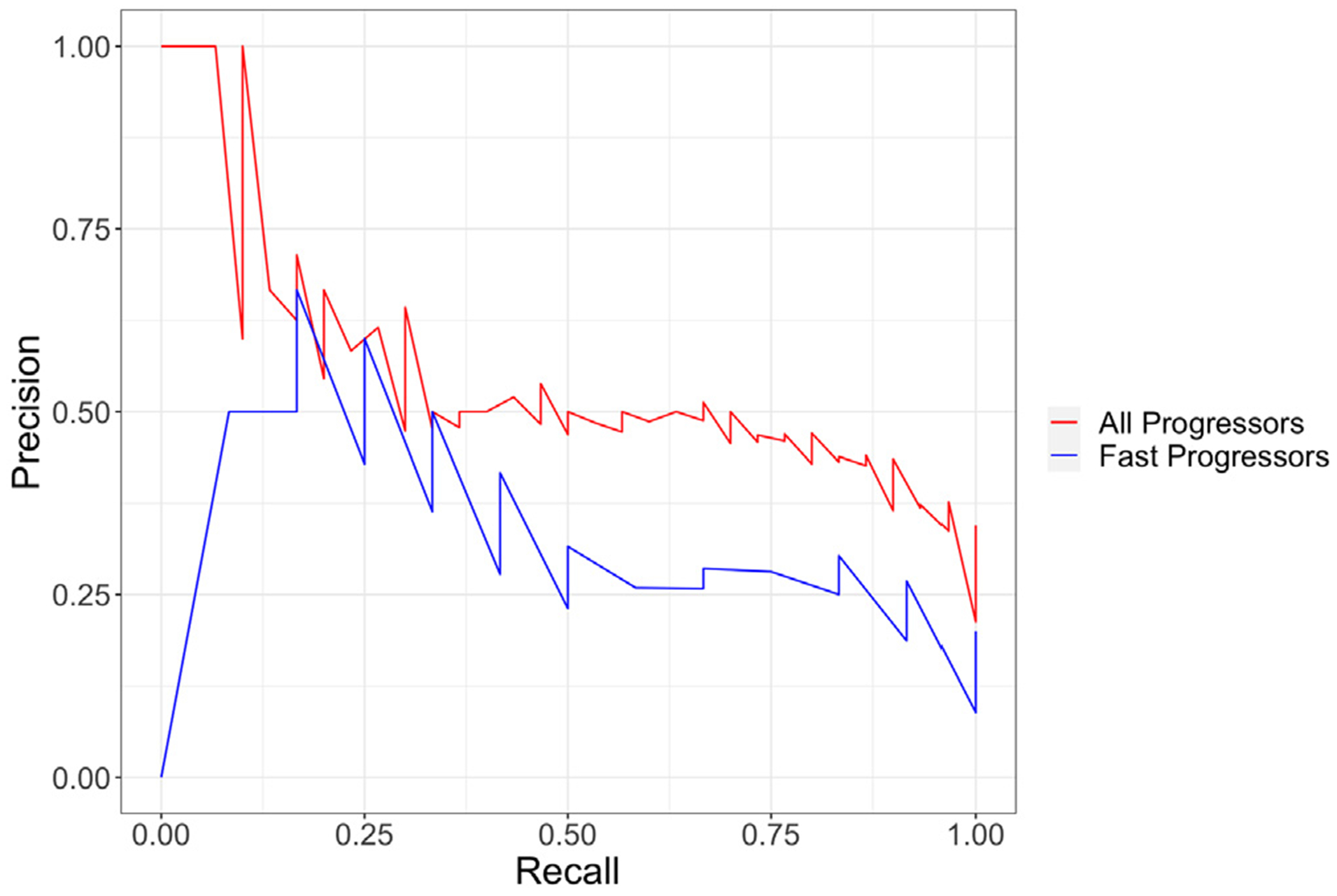
The precision-recall curve of the best-performing deep learning model for all progressors and fast-progressing eyes. The area under the precision-recall curve was 0.558 and 0.383, respectively.

Figure 3 represents the precision-recall curve of the model utilizing ODP and RNFL and macular thickness measurements for all progressors and fast-progressing eyes. For all progressors, the precision (95% CI) and recall were 46.0% (36.0%-59.0%) and 90.0% (73.0%-100.0%), and the AU-PRC was 0.558 (0.385-0.733), Table 2. On the other hand, for the fast progressors, the precision, recall, and AU-PRC were 29.0% (18.0%-42.0%), 91.0% (64.0%-95.0%), and 0.383 (0.182-0.681), respectively (Table 3).

**FIGURE 4.**
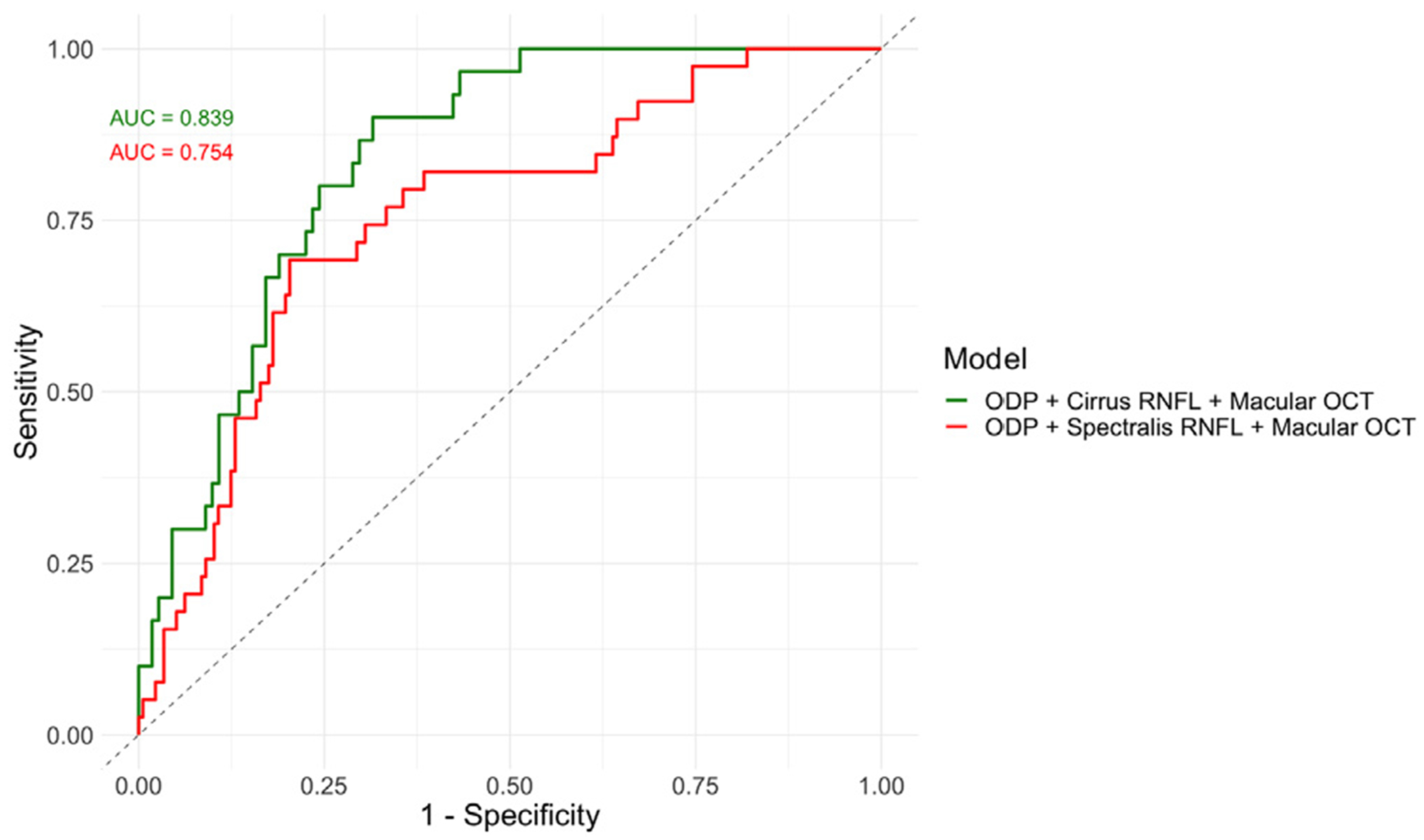
Comparison of the receiver operating characteristic (ROC) curves for the models incorporating all three structural modalities. Green curve, the cohort of eyes with which training and testing were done (original cohort); optical coherence tomography (OCT) was done with Cirrus OCT in this cohort. Red curve, external validation cohort ROC curve; OCT imaging was done with Spectralis OCT.

The DL models were tested on a dataset of eyes in which RNFL and macular OCT were acquired with the Spectralis OCT. The performance of all the models was slightly lower than their performance on the original dataset, in which the OCT imaging was carried out with Cirrus HD-OCT. The AUC for the model incorporating all the structural modalities on the validation dataset was 0.754 (0.671-0.837), which, although lower than the AUC of the same model of the original dataset, was not statistically significant (*P* = .122). The most complex model performed better than most of the other simpler models (Table 4). We also performed logistic regression and Lasso regression models on this dataset; the AUC (95% CI) of the two models was 0.576 (0.480-0.672) and 0.608 (0.515-0.702), which were significantly lower than the DL model utilizing all structural modalities (*P* = .006 and 0.002, respectively). Similar to the original dataset, two glaucoma specialists were asked to predict the likelihood of future glaucoma progression after they were provided the demographic and clinical data and structural findings from this dataset; the AUC (95% CI) of the clinicians’ grading were 0.438 (0.356-0.520) and 0.531 (0.444-0.681), which were significantly lower than the AUC for the DL model tested on the same dataset (*P* < .001). The Cohen Kappa agreement for the performance between the two clinicians was 0.07 (0.04-0.11). Figure 4 demonstrates the ROC curves of the best-performing model based on the original dataset and the validation dataset. The pAUC of all the models as implemented on the external validation dataset is provided in Supplementary Table 2.

Supplementary Figures 1 to 3 demonstrate examples of 3 eyes classified by both the progression criteria and the DL model for each group of stable, progressing, and rapidly progressing eyes, respectively. For each eye, the demographic data, the optic disc photograph, and RNFL and macular thickness maps at baseline are provided. The progressing and fast-progressing eyes demonstrate substantial structural glaucomatous damage at baseline. Of note, although in these examples the OCT thickness maps are displayed, only numerical OCT thickness measurements were put into the DL model.

## DISCUSSION

We designed an AI-based prognostic model to predict future functional glaucoma progression from a combination of various baseline structural data, along with clinical and demographic information. We found that the model that incorporated all three types of structural data, ie, ODP and RNFL and macular OCT images, demonstrated the highest performance with an AUC of 0.839 and accuracy of 76.0%. The performance of this model was significantly higher than logistic regression, the Lasso regression model, and clinical classification by glaucoma specialists. Models with various pairs of structural data also showed an acceptable performance with AUCs between 0.664 and 0.811. Most of the DL models had a higher AUC magnitude when implemented to predict fast-progressing eyes, defined as MD rates of change of less than −1 dB/y; the AUC and accuracy of the most complex model were 0.869 and 77.0%, respectively. We validated the DL model on a separate cohort of eyes in which a different OCT device was used to acquire RNFL and macular images. The DL models demonstrated similar performance when applied to the cohort in which OCT was done with Spectralis OCT. Our findings underscore the significance of developing robust prognostic models that integrate structural, clinical, and demographic data to enhance prediction of future glaucoma progression. Such models have the potential to transform glaucoma management by enabling tailored monitoring and treatment plans, including earlier intervention, with the aim of preserving vision and improving the quality of life for glaucoma patients.

Landmark glaucoma studies such as the Ocular Hypertension Treatment Study, Early Manifest Glaucoma Trial (EGMT) and Collaborative Normal-Tension Glaucoma Study (CNTGS), investigated factors associated with glaucoma progression as either primary or secondary out-comes.^9,11,55–57^ Older age, African American race, higher IOP, thinner CCT, larger vertical cup/disc ratio and higher PSD predicted conversion to glaucoma in OHTS.^9^ Thinner CCT and lower diastolic blood pressure forecast VF deterioration in EGMT.^56^ Female sex, a history of migraine, and optic disc hemorrhages were found to be associated with a higher risk of glaucoma progression in CNTGS.^57^ The main structural variable that was investigated in these studies was the cup-to-disc ratio. Most such studies used Cox’s regression model. More recent studies have reported the utility of baseline and longitudinal OCT measurements for the prediction of glaucoma progression.^38, 40, 58, 59^ Our study is the first to combine widely used structural data, including disc photographs, and RNFL and macular OCT data with relevant clinical and demographic information, utilizing an AI platform to predict future VF progression. Incorporating the above structural parameters into prognostic models is expected to enhance DL models’ predictive accuracy by offering objective and quantifiable markers of disease state. Additionally, including clinical variables such as IOP, CCT, VF parameters, and demographic factors like age, gender, and race could improve DL models’ predictive power by accounting for individual variability in disease susceptibility.

An important aspect of this study was the comparison of various combinations of structural data with regard to prediction of future glaucoma progression. When only one structural measure was combined with clinical and demographic data, the AUC for predicting glaucoma progression ranged from 0.664 to 0.781. With two structural measures, prediction improved; incorporating all three structural data elements into the model provided the highest and most clinically relevant performance for forecasting glaucoma progression. As the three structural modalities used in this study convey somewhat different signals regarding the health of RGCs, ie, the neuroretinal rim, RNFL, and macular thickness, it would be expected that adding them as separate pieces of the input data for the DL model would potentially help enhance model performance.^35, 60^ Therefore, this multimodal approach allows for the “perception” of more subtle structural signals that may not be apparent with a single structural measure alone, thereby improving the sensitivity and specificity of disease progression predictions. The AU-PRC provides a more practical metric for the performance of AI models regarding forecasting glaucoma deterioration; the AU-PRC for the most complex model was 0.558 with 46% precision (positive predictive value) and 90% recall (sensitivity), and given the low prevalence of progressing eyes, these values are promising for predicting glaucoma progression. Combining structural data can mitigate the limitations inherent in each imaging modality, leading to more robust and reliable assessment tools. This holistic integration of structural measures seems to facilitate earlier detection of disease progression, in turn allowing more accurate monitoring of disease trajectory and tailoring of therapeutic strategies to individual patient profiles, ultimately contributing to better clinical outcomes.

In this study, the DL model significantly outperformed clinical classification by two experienced glaucoma specialists, achieving higher AUC values when applied to the same multimodal data. While the specialists’ performance was above chance, their AUCs (0.629 and 0.680) highlight the inherent challenges of predicting future glaucoma progression using structural, functional, and demographic information alone. The modest interobserver agreement (Cohen’s kappa = 0.28, 95% CI: 0.12-0.44) further underscores the variability and subjectivity in expert judgment, even among subspecialists reviewing identical datasets. These findings suggest that DL models may provide more consistent and reproducible prognostic assessments compared to human evaluation, particularly in scenarios where progression is less prominent and difficult to detect clinically. Importantly, rather than replacing clinician judgment, such models could serve as valuable adjuncts, standardizing risk assessment and helping guide clinical decision-making in glaucoma management.

All of the DL models demonstrated high AUCs for prediction of glaucoma progression in fast-progressing glaucoma eyes. A rate of change of −1 dB/y or faster has been frequently used to define fast functional progression.^61^ The AUC of the most complex model for detection of fast progression was 0.869 (95% CI: 0.792-0.947) and the AUCs of the less complex models were all greater than 0.731 except for the model evaluating only demographic variables, for which the AUC was 0.667. However, given the unbalanced nature of the data regarding fast progression (9.8%), AU-PRC provides a better metric for the practical utility of the AI model. The AU-PRC for this subgroup was 0.383 with a high recall (100%) and low precision (29%). While highly sensitive, our most complex multimodal model had a low positive predictive value. Improving the performance of the model forecasting fast progression is limited by the generally small number of such eyes in most glaucoma cohorts. Early identification of rapid progression in glaucoma eyes is crucial so as to prevent significant vision loss, as these patients require timely and more aggressive intervention. Certain groups of patients are particularly prone to faster glaucoma progression, including those with higher baseline IOP, advanced age, African or Hispanic ethnicity, a family history of glaucoma, and those with thinner CCT.^13,62^ Forecasting rapid progression in high-risk patients would enable clinicians to implement more intensive monitoring and therapeutic strategies.

Establishing generalizability is essential with any model or algorithm designed to be commonly used in the clinical setting. While clinical and demographic data and disc photographs are uniformly collected or acquired, various OCT devices are used around the world for imaging the RNFL or macular region. Each OCT device uses a proprietary segmentation module, and therefore, RNFL or macular measurements are not necessarily comparable among various OCT devices.^63–65^ Therefore, we set out to establish that our algorithm’s performance does not suffer when data from different OCT devices are used. Cirrus HD-OCT and Spectralis OCT are the most commonly used devices in the US. We tested the DL model, which was originally trained on a cohort of eyes that had their imaging carried out with Cirrus HD-OCT, on a separate dataset of eyes in which OCT imaging was done with Spectralis OCT. Model performance, when used on this cohort, was lower compared to the original cohort, although this was not statistically significant. One likely explanation for this finding is the inherent variability in how different OCT platforms acquire and measure RNFL and macular thickness measurements. For instance, Cirrus HD-OCT reports GCIPL thickness in a 4.0 × 4.8-mm elliptical region centered on the fovea, whereas Spectralis OCT measures macular thickness on an 8 × 8 mm square using the Posterior Pole Algorithm. To harmonize the datasets, postprocessing adjustments were applied to the raw Spectralis RNFL and macular outputs to approximate the Cirrus HD-OCT format. These steps were necessary because the deep-learning model was trained exclusively on Cirrus-derived data; therefore, aligning Spectralis outputs to the Cirrus structure allowed the trained network to utilize the Spectralis OCT data in the second database. Despite these harmonization efforts, intrinsic differences in device acquisition protocols, segmentation algorithms, and scanning geometry may have contributed to the reduced model performance observed in the Spectralis cohort.^66^ To overcome this limitation, future work should focus on training the model directly on raw OCT data or developing multidevice training strategies that incorporate heterogeneous OCT inputs, thereby improving crossplatform generalizability.

In our study, we defined functional glaucoma progression using the MD slope derived from standard automated perimetry, a methodology commonly employed in longitudinal glaucoma research. We specifically selected fixed 5-year time windows for linear regression to help mitigate the potential nonlinearity of MD change over longer follow-up periods. Prior work has suggested that VF decline may not always follow a uniform linear trend across extended periods, particularly as patients approach advanced disease stages or encounter treatment intensification. By applying consistent 5-year intervals across all eyes, our approach ensured a standardized and comparable definition of functional progression while reducing bias associated with nonlinear long-term trajectories. Although nonlinear models may provide an alternative means of characterizing VF change in future studies, the fixed-interval linear approach remains well-validated and clinically meaningful for assessing functional deterioration over time.^67,68^

The limitations of our study must be considered in light of the methods. We used the rate of MD change to define functional glaucoma progression. There is no gold standard method for detection of functional glaucoma progression. Some studies have argued that pointwise trend-based methods are able to detect glaucoma functional progression earlier in the course of the disease.^69^ However, the MD rate has been demonstrated to be a valid endpoint for determining glaucoma progression in clinical and research settings.^70^ Global rates of change such as MD rates minimize longitudinal measurement variability due to the averaging of noise.^71^ The macular OCT measurements derived from Cirrus and Spectralis OCTs do not exactly correspond to each other although we made them as similar as possible. The final models, however, performed as well when using Spectralis macular and RNFL data. This suggests that our algorithm may perform adequately when OCT data from other available devices are used as input. An important limitation of our study, as with most retrospective studies of glaucoma progression, is the potential for confounding by indication. Eyes that demonstrate progression are often treated more aggressively, which may in turn slow the subsequent rate of progression and obscure the true natural history of the disease. To address this bias, we incorporated data on treatment intensification, including increases in the number of IOP-lowering medications as well as any laser or surgical interventions during follow-up. However, these treatment variables also have limitations, as their reliability may be influenced by both internal and external factors such as patient demographics and adherence to prescribed therapies. Notably, a review has reported that only about 50% of glaucoma patients are compliant with their IOP-lowering eye drops.^72^ To account for internal factors, baseline demographic variables were included in the DL model, while to address variability in patient adherence, IOP during follow-up was also added as a model input. Incorporating these additional data elements into the DL model may help mitigate the effect of confounding by indication and improve the robustness of the model in predicting glaucoma progression.

Future clinical deployment of this model will require seamless integration into routine ophthalmic workflows. Embedding the algorithm within electronic health record systems will allow automated extraction of structural and clinical data, real-time generation of individualized progression-risk estimates, and intuitive visualization for clinicians at the point of care. Moreover, incorporating longitudinal imaging and clinical information through continuous data streams could enable adaptive model refinement and dynamic updating of risk predictions. Prospective validation in real-world settings, usability testing, and pilot deployment studies will be essential next steps to evaluate clinical utility, workflow efficiency, and potential impact on treatment decisions. Ultimately, thoughtful integration with electronic health record platforms and clinical decision-support systems will be fundamental for translating this AI tool into a scalable, patient-centered solution for glaucoma management.

In conclusion, our novel AI-based prognostic model is able to predict future glaucoma progression from baseline disc photographs, RNFL and macular OCT measurements, and demographic and clinical data with clinically relevant performance. The positive predictive value of the model was low when implemented to predict fast-progressing glaucoma eyes. The model demonstrates generalizability when it was implemented with a different OCT device’s data. This DL model can help clinicians in detecting patients at high risk of glaucoma progression and improve monitoring of glaucoma patients in the clinical and research setting.

## Supplementary Material

1

2

3

4

5

6

Supplemental Material available at AJO.com.

## Figures and Tables

**TABLE 1.** Demographic and Clinical Characteristics of Study Participants.

	Eyes = 1599; Patients = 908		
	Stable (Eyes = 1200)	Progressing (Eyes = 399)	P Value
*Age at baseline (y)*	58.6 (11.8)	61.3 (9.2)	< .001
*Gender*			
*Female*	402	131	.797
*Male*	284	91	
*Race*			
*White*	422	136	.784
*African American*	56	18	
*Asian*	79	34	
*Hispanic*	100	26	
*Other*	28	8	
*Follow-up time (y)*	9.7 (4.6)	11.4 (4.7)	< .001
*Number of VF exams, median (IQR)*	9 (7-13)	14 (10-21)	< .001
*Baseline MD (dB)*	−3.3 (4.9)	−4.1 (4.9)	.002
*Baseline PSD*	3.8 (3.6)	4.7 (3.7)	< .001
*Baseline IOP (mm Hg)*	15.3 (2.9)	15.0 (3.0)	.050
*Baseline CCT (μm)*	559.7 (37.8)	551.5 (41.6)	< .001
*Global RNFL thickness at baseline (μm)*	79.0 (14.9)	70.2 (13.5)	< .001
*Global macular thickness at baseline (μm)*	68.2 (12.9)	61.8 (12.8)	< .001

CCT = central cornea thinness; IOP = intraocular pressure; IQR = interquartile range; MD = mean deviation; PSD = pattern standard deviation; RNFL = retinal nerve fiber layer.

**TABLE 2.** The Area Under Receiver Operating Characteristics, Sensitivity, Specificity, Accuracy, and Precision-Recall Area Under Receiver Characteristic Curve of Different Deep Learning Models That Evaluated All Possible Combinations of Structural Data Along With Demographic Information, and the Model With Solely Demographics.

Input Data	AUC	P Value^a^	Sensitivity	Specificity	Accuracy	PRAUC	P Value^a^
*Only demographics*	0.657 (0.560-0.754)	<.001	80.0% (63.0%-93.0%)	53.0% (44.0%-62.0%)	59.0% (51.0%-67.0%)	0.285 (0.200-0.428)	< .001
*Baseline ODP*	0.664 (0.550-0.777)	.003	70.0% (53.0%-87.0%)	65.0% (56.0%-74.0%)	66.0% (58.0%-74.0%)	0.336 (0.222-0.522)	.022
*Baseline RNFL*	0.732 (0.632-0.831)	.001	83.0% (70.0%-97.0%)	66.0% (57.0%-75.0%)	70.0% (62.0%-77.0%)	0.397 (0.270-0.571)	.010
*Baseline macula*	0.781 (0.690-0.871)	.151	80.0% (63.0%-93.0%)	71.0% (62.0%-79.0%)	73.0% (66.0%-80.0%)	0.383 (0.257-0.574)	.022
*Baseline* *ODP* + *RNFL*	0.787 (0.696-0.878)	.130	73.0% (57.0%-87.0%)	72.0% (64.0%-80.0%)	72.0% (65.0%-79.0%)	0.507 (0.327-0.687)	.595
*Baseline* *ODP* + *macula*	0.789 (0.704-0.874)	.239	87.0% (70.0%-100.0%)	75.0% (55.0%-86.0%)	77.0% (64.0%-84.0%)	0.416 (0.292-0.600)	.124
*Baseline* *RNFL* + *macula*	0.811 (0.738-0.884)	.589	90.0% (73.0%-100.0%)	71.0% (56.0%-86.0%)	75.0% (65.0%-84.0%)	0.432 (0.311-0.621)	.137
*Baseline* *ODP* + *RNFL* + *macula*	0.839 (0.771-0.906)	—	90.0% (73.0%-100.0%)	71.0% (53.0%-86.0%)	76.0% (62.0%-85.0%)	0.558 (0.385-0.733)	—

For all of the models, the demographic and clinical data were considered as input. The model evaluating all three structural data (last row) demonstrated the highest performance.

AUC = area under receiver characteristic curve; ODP = optic disc photo; PRAUC = precision-recall area under receiver characteristic curve; RNFL = retinal nerve fiber layer.

a *P* values were from the comparison of each model with the model evaluating all three structural data.

**TABLE 3.** The Performance of the Deep Learning Models for Predicting Future Functional Glaucoma Progression for the Fast-Progressing Eyes (Mean Deviation Rates <−1 dB/Y).

Input Data	AUC	P Value^a^	Sensitivity	Specificity	Accuracy	PRAUC	P Value^a^
*Only demographics*	0.667 (0.539-0.795)	.010	33% (13.0%-60.0%)	79% (71.0%-85.0%)	75.0% (67.0%-82.0%)	0.135 (0.073-0.254)	.012
*Baseline ODP*	0.731 (0.638-0.823)	.015	66.0% (39.0%-86.0%)	66.0% (57.0%-74.0%)	66.0% (58.0%-74.0%)	0.154 (0.084-0.272)	.007
*Baseline RNFL*	0.781 (0.660-0.901)	.090	83.0% (55.0%-95.0%)	60.0% (51.0%-68.0%)	62.0% (53.0%-70.0%)	0.307 (0.124-0.605)	.599
*Baseline macula*	0.772 (0.651-0.893)	.065	91.0% (64.0%-98%)	53.0% (44.0%-62.0%)	57.0% (48.0%-65.0%)	0.264 (0.116-0.532)	.240
*Baseline ODP* + *RNFL*	0.841 (0.758-0.924)	.291	91.0% (64.0%-98.0%)	73.0% (75.0%-80.0%)	75.0% (67.0%-82.0%)	0.337 (0.142-0.585)	.553
*Baseline ODP* + *macula*	0.754 (0.605-0.902)	.115	75.0% (46.0%-91.0%)	73.0% (65.0%-80.0%)	74.0% (66.0%-80.0%)	0.325 (0.133-0.633)	.641
*Baseline RNFL* + *macula*	0.829 (0.746-0.913)	.490	92.0% (75.0%-100.0%)	68.0% (60.0%-76.0%)	70.0% (63.0%-78.0%)	0.249 (0.110-0.527)	.517
*Baseline ODP* + *RNFL* + *macula*	0.869 (0.792-0.947)	—	91.0% (64.0%-95.0%)	75.0% (67.0%-82.0%)	77.0% (69.0%-83.0%)	0.383 (0.182-0.681)	—

For all of the models, the demographic and clinical data were considered as input.

AUC = area under receiver characteristic curve; ODP = optic disc photo; PRAUC = precision-recall area under receiver characteristic curve; RNFL = retinal nerve fiber layer.

a *P* values were from the comparison of each model with the model evaluating all three structural data.

**TABLE 4.** The Results of Validating All Seven Deep Learning Models on the Separate Dataset of Eyes That Their Retinal Nerve Fiber Layer and Macular OCT Were Performed With Spectralis OCT.

Input Data	AUC	P Value^a^	Sensitivity	Specificity	Accuracy	PRAUC	P Value^a^
*Only demographics*	0.502 (0.411-0.593)	<.001	89.0% (76.0%-96.0%)	26.0% (20.0%-33.0%)	37.0% (31.0%-44.0%)	0.165 (0.114-0.231)	< .001
*Baseline ODP*	0.719 (0.635-0.803)	.530	71.0% (56.0%-83.0%)	68.0% (60.0%-74.0%)	68.0% (62.0%-74.0%)	0.348 (0.240-0.506)	.562
*Baseline RNFL*	0.678 (0.571-0.785)	.006	61.0% (45.0%-75.0%)	74.0% (67.0%-80.0%)	71.0% (65.0%-77.0%)	0.338 (0.235-0.489)	.296
*Baseline macula*	0.700 (0.614-0.786)	.130	53.0% (38.0%-68%)	77.0% (70.0%-83.0%)	73.0% (68.0%-78.0%)	0.308 (0.222-0.455)	.182
*Baseline ODP* + *RNFL*	0.744 (0.663-0.825)	.494	79.0% (61.0%-78.0%)	69.0% (62.0%-78.0%)	71.0% (64.0%-76.0%)	0.349 (0.246-0.492)	.149
*Baseline* *ODP* + *macula*	0.698 (0.610-0.787)	.128	56.0% (41.0%-70.0%)	77.0% (70.0%-82.0%)	73.0% (67.0%-78.0%)	0.312 (0.222-0.460)	.158
*Baseline* *RNFL* + *macula*	0.710 (0.618-0.802)	.485	66.0% (51.0%-79.0%)	72.0% (65.0%-78.0%)	71.0% (65.0%-77.0%)	0.327 (0.231-0.479)	.115
*Baseline* *ODP* + *RNFL* + *macula*	0.754 (0.671-0.837)	—	69.0% (53.0%-81.0%)	79.0% (73.0%-84.0%)	77.0% (71.0%-82.0%)	0.382 (0.250-0.532)	

For all of the models, the demographic and clinical data were considered as input.

AUC = area under receiver characteristic curve; ODP = optic disc photo; PRAUC = precision-recall area under receiver characteristic curve; RNFL = retinal nerve fiber layer.

a *P* values were from the comparison of each model with the model evaluating all three structural data.
