# Supplementary material for: An Artificial Intelligence-Based Prognostic Model for Prediction of Functional Glaucoma Progression From Clinical and Structural Data
Source: Am J Ophthalmol. Author manuscript; Available in PMC 2026 Jul 17. (PMC13379235; doi:10.1016/j.ajo.2025.12.026)
Supplement: 1 [file NIHMS2189849-supplement-1.pdf]

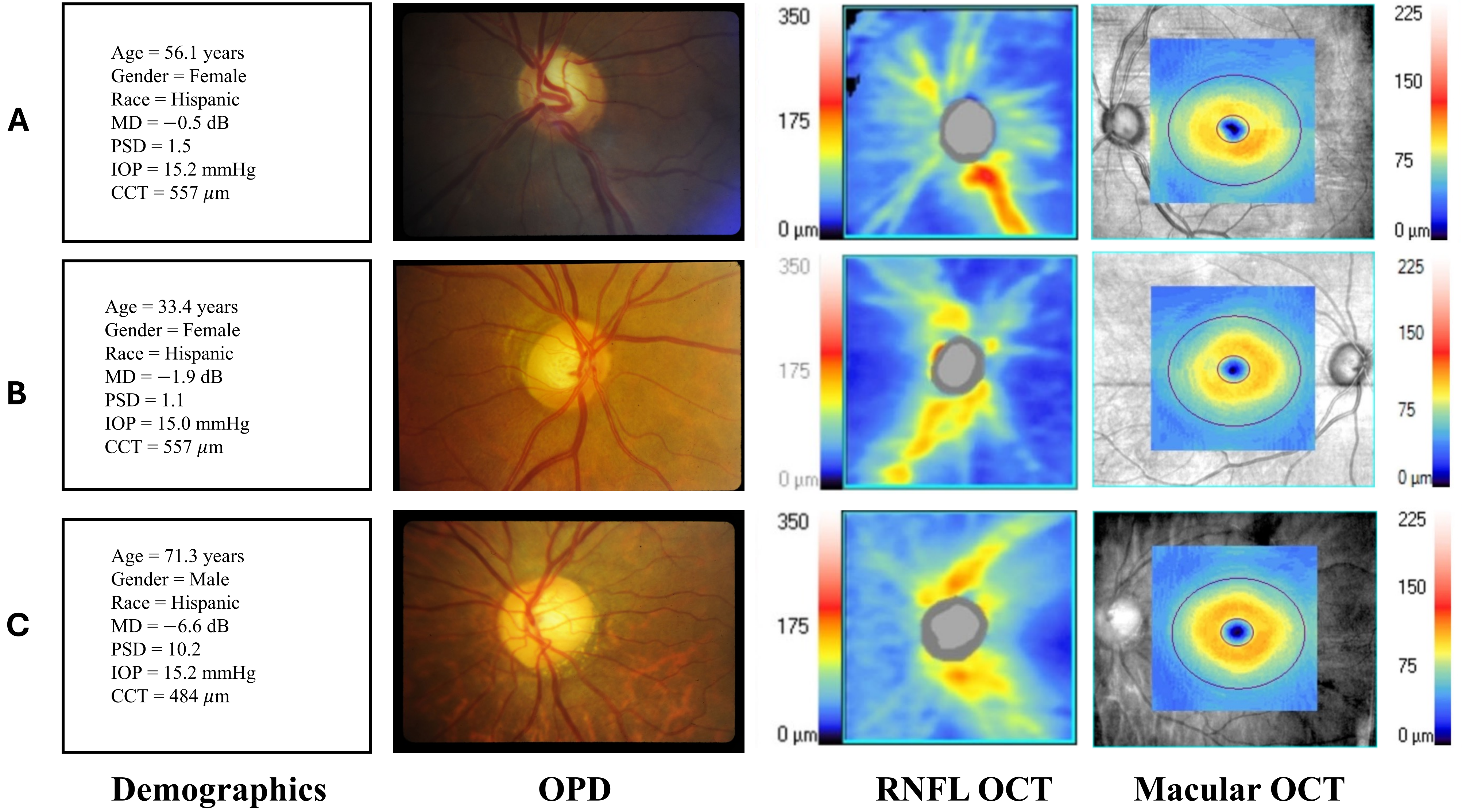
A
Age = 56.1 years
Gender = Female
Race = Hispanic
MD = −0.5 dB
PSD = 1.5
IOP = 15.2 mmHg
CCT = 557 μm
B
Age = 33.4 years
Gender = Female
Race = Hispanic
MD = −1.9 dB
PSD = 1.1
IOP = 15.0 mmHg
CCT = 557 μm
C
Age = 71.3 years
Gender = Male
Race = Hispanic
MD = −6.6 dB
PSD = 10.2
IOP = 15.2 mmHg
CCT = 484 μm
350
175
0 μm
225
150
75
0 μm
Demographics
OPD
RNFL OCT
Macular OCT
