# Supplementary material for: An Artificial Intelligence-Based Prognostic Model for Prediction of Functional Glaucoma Progression From Clinical and Structural Data
Source: Am J Ophthalmol. Author manuscript; Available in PMC 2026 Jul 17. (PMC13379235; doi:10.1016/j.ajo.2025.12.026)
Supplement: 2 [file NIHMS2189849-supplement-2.pdf]

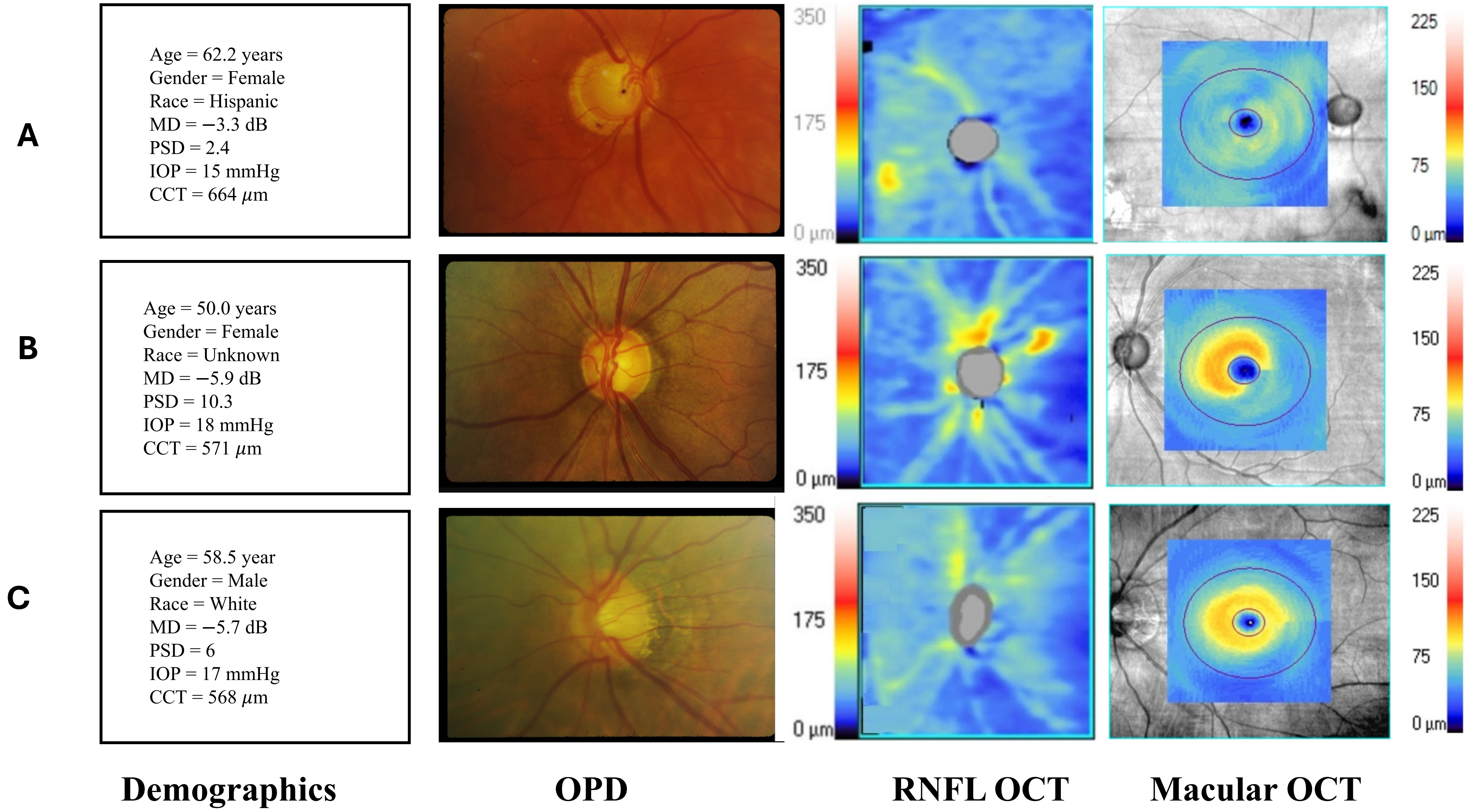
A
Age = 62.2 years
Gender = Female
Race = Hispanic
MD = −3.3 dB
PSD = 2.4
IOP = 15 mmHg
CCT = 664 μm
350
175
0 μm
225
150
75
0 μm
B
Age = 50.0 years
Gender = Female
Race = Unknown
MD = −5.9 dB
PSD = 10.3
IOP = 18 mmHg
CCT = 571 μm
350
175
0 μm
225
150
75
0 μm
C
Age = 58.5 year
Gender = Male
Race = White
MD = −5.7 dB
PSD = 6
IOP = 17 mmHg
CCT = 568 μm
350
175
0 μm
225
150
75
0 μm
Demographics
OPD
RNFL OCT
Macular OCT
