# Supplementary material for: An Artificial Intelligence-Based Prognostic Model for Prediction of Functional Glaucoma Progression From Clinical and Structural Data
Source: Am J Ophthalmol. Author manuscript; Available in PMC 2026 Jul 17. (PMC13379235; doi:10.1016/j.ajo.2025.12.026)
Supplement: 3 [file NIHMS2189849-supplement-3.pdf]

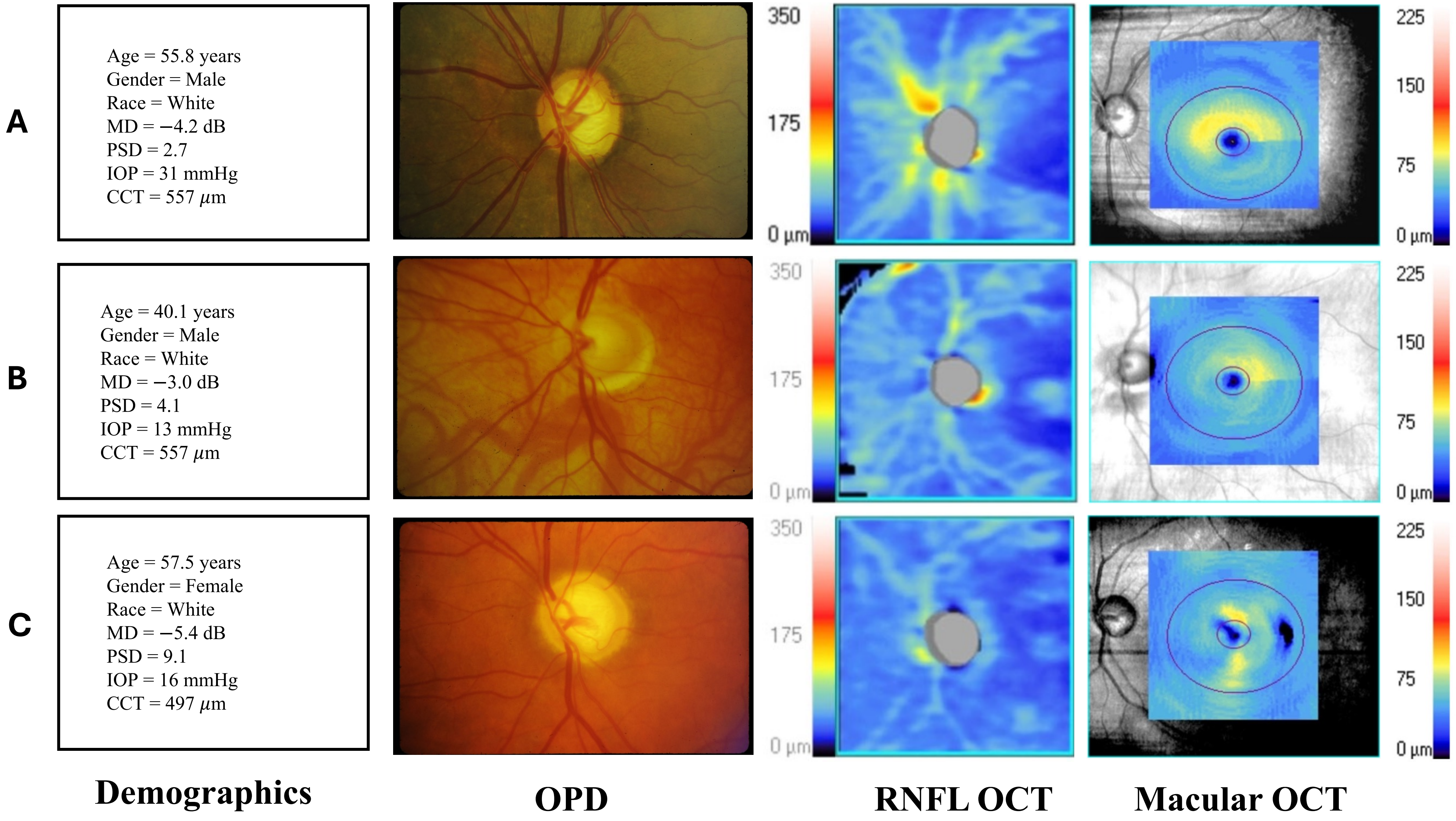
A
Age = 55.8 years
Gender = Male
Race = White
MD = −4.2 dB
PSD = 2.7
IOP = 31 mmHg
CCT = 557 μm
B
Age = 40.1 years
Gender = Male
Race = White
MD = −3.0 dB
PSD = 4.1
IOP = 13 mmHg
CCT = 557 μm
C
Age = 57.5 years
Gender = Female
Race = White
MD = −5.4 dB
PSD = 9.1
IOP = 16 mmHg
CCT = 497 μm
350
175
0 μm
225
150
75
0 μm
Demographics
OPD
RNFL OCT
Macular OCT
