# Supplementary material for: An Artificial Intelligence-Based Prognostic Model for Prediction of Functional Glaucoma Progression From Clinical and Structural Data
Source: Am J Ophthalmol. Author manuscript; Available in PMC 2026 Jul 17. (PMC13379235; doi:10.1016/j.ajo.2025.12.026)
Supplement: 4 [file NIHMS2189849-supplement-4.pdf]

Supplementary Table 1. TRIPOD+AI checklist for the reporting of prediction model studies

Section/Topic	Item No	Checklist Item	Reported on Page/Line	Reported on Section/Paragraph
Title	1	Identify the study as developing or evaluating the performance of a multivariable prediction model, the target population, and the outcome to be predicted	Page 1, Line 1-3	Title Page
Abstract	2	See TRIPOD+AI for Abstracts checklist	Abstract	Abstract section
Introduction - Target population	3b	Describe the target population and intended purpose of the prediction model	Page 3, Lines 70-73	Introduction, Paragraph 4
Introduction - Health inequalities	3c	Describe any known health inequalities between sociodemographic groups	N/A	Not covered; study describes demographics but not inequalities
Introduction - Objectives	4	Specify the study objectives, including whether the study describes development or validation of a prediction model	Page 3, Lines 69-73	Introduction, Paragraph 4
Methods - Data sources	5a	Describe the sources of data and representativeness	Page 4, Lines 75-91	Methods, Paragraphs 1-2
Methods - Dates	5b	Specify dates of participant data, accrual, and follow-up	Page 4, Lines 75-80	Methods, Paragraph 1
Methods - Setting	6a	Specify key elements of study setting and centres	Page 4, Lines 75-80	Methods, Paragraph 1
Methods - Eligibility criteria	6b	Describe eligibility criteria for participants	Page 4, Lines 85-91	Methods, Paragraphs 2
Methods - Treatments	6c	Give details of treatments received and how handled	Page 6, Lines 127-130	Methods, Paragraph 6
Methods - Data preparation	7	Describe data pre-processing and quality checking	Page 7, Lines 146-152	Methods, Paragraph 8
Methods - Outcome definition	8a	Define outcome predicted and time horizon	Page 8-9, Lines 177-189	Methods, Paragraph 11

Methods - Outcome assessor	8b	If subjective interpretation required, describe assessors	N/A	Not applicable; outcome defined algorithmically
Methods - Outcome blinding	8c	Report any blinding of outcome assessment	N/A	Not performed; outcome statistical
Methods - Predictors choice	9a	Describe choice of initial predictors	Page 4-6, Lines 92-130	Methods, Paragraph 3-6
Methods - Predictors definition	9b	Define all predictors, measurement details	Page 4-6, Lines 92-130	Methods, Paragraph 3-6
Methods - Predictor assessor	9c	If predictor interpretation subjective, describe assessors	N/A	Not applicable; predictors algorithmic
Methods - Sample size	10	Explain how sample size was determined and sufficiency	N/A	Large data study
Methods - Missing data	11	Describe handling of missing data	N/A	There was no missing data
Methods - Data usage	12a	Describe how data used in analysis (development/evaluation)	Pages 7-8, Lines 145-176	Methods, Paragraphs 8-10
Methods - Predictor handling	12b	Describe handling of predictors (form, rescaling, etc.)	Page 7-8, Lines 153-170	Methods, Paragraph 9
Methods - Model building	12c	Specify type of model, rationale, hyperparameters, internal validation	Page 7-8, Lines 153-170	Methods, Paragraphs 9
Methods - Heterogeneity	12d	Describe handling of heterogeneity across clusters	N/A	Not applicable; single-centre study
Methods - Performance measures	12e	Specify measures/plots to evaluate performance	Page 8-9, Lines 177-189	Methods, Paragraph 11
Methods - Model updating	12f	Describe model updating if performed	N/A	Not performed
Methods - Prediction calculation	12g	Describe how predictions calculated	Page 8-9, Lines 177-189	Methods, Paragraph 11
Methods - Class imbalance	13	Describe handling of class imbalance	Page 8, Lines 171-176	Methods, Paragraph 10
Methods - Fairness	14	Describe approaches to address fairness	N/A	Not addressed explicitly
Methods - Model output	15	Specify model output (probabilities/classification)	Page 8-9, Lines 177-189	Methods, Paragraph 11
Methods - Training vs evaluation	16	Identify differences between development/evaluation datasets	Page 7, Lines 146-152	Methods, Paragraph 8
Methods - Ethics	17	IRB approval and consent	Page 4, Lines 76-77	Methods, Paragraph 1

Funding	18a	Source of funding and role of funders	Page 23, Line 459-460	Acknowledgements, Funding
Conflicts of interest	18b	Declare conflicts and disclosures	Page 23, Line 462	Footnote/Competing Interests
Protocol	18c	Indicate where study protocol can be accessed/state none	N/A	No protocol prepared
Registration	18d	Provide registration info	N/A	Not registered
Data sharing	18e	Details of study data availability	Page 22	Statement: Available on request
Code sharing	18f	Details of code availability	Page 22	Statement: Available on request
Patient involvement	19	Provide details of patient/public involvement	N/A	None
Results - Participant flow	20a	Describe flow of participants through study	Page 12, Lines 239-248;	Results, Paragraph 1
Results - Participant characteristics	20b	Report baseline characteristics overall/by groups	Page 12, Lines 239-248; Table 1	Results, Paragraph 1 + Table 1
Results - Development vs evaluation comparison	20c	Compare development vs evaluation data	Page 12-13, Lines 249-270	Results, Paragraph 2
Results - Numbers in analyses	21	Specify numbers in each analysis	Page 12-14, Lines 249-291	Results, Paragraphs 2-5
Results - Model specification	22	Provide full model details	Figure 1	Methods/Results Figures
Results - Model performance	23a	Report performance estimates with CI	Page 12-14, Lines 249-291	Results, Paragraphs 2-5, Tables 2-4
Results - Heterogeneity of performance	23b	Report heterogeneity in performance across clusters	N/A	Not done; single-centre
Results - Model updating	24	Report results from model updating	N/A	Not done
Discussion - Interpretation	25	Interpret results, fairness, context with prior studies	Page 15-19 lines 300-399	Discussion, Paragraphs 1-6
Discussion - Limitations	26	Discuss limitations and biases	Page 19-20 lines 400-425	Discussion, Paragraph 7
Discussion - Usability (data quality)	27a	Describe handling of poor-quality input data in practice	N/A	Not discussed explicitly
Discussion - Usability (user expertise)	27b	Specify user expertise required for model use	Page 17-18 lines 357-369	Discussion, paragraph 4
Discussion - Next steps	27c	Discuss next steps, applicability, generalisability	Page 22	Conclusions, Paragraph 1
