# Supplementary material for: An Artificial Intelligence-Based Prognostic Model for Prediction of Functional Glaucoma Progression From Clinical and Structural Data
Source: Am J Ophthalmol. Author manuscript; Available in PMC 2026 Jul 17. (PMC13379235; doi:10.1016/j.ajo.2025.12.026)
Supplement: 5 [file NIHMS2189849-supplement-5.pdf]

Supplementary Table 2. The partial area under the receiver operating characteristics curves (for specificity >90%) for different deep learning models incorporating all possible combinations of structural data along with demographic/clinical information and the model with only demographic/clinical data. The criteria for all progressors and fast progressors were MD rates <0 dB/year and <−1 dB/year, respectively with a confirmation and significant rates of change for the entire series. For the external validation dataset optical coherence tomography (OCT) scans were acquired with Spectralis OCT instead of Cirrus OCT for the original dataset. For all of the models, the demographic and clinical data were considered as input.

Input	*Formal progressors*		*Fast progressors*		*External validation*	
	Partial AUC	*P-value*	*Partial AUC*	*P-value*	*Partial AUC*	*P-value*
Only Demographics	0.003 (0-0.013)	0.030	0.001 (0-0121)	0.019	0.00 (0-0.004	0.026
Baseline ODP	0.010 (0.002-0.023)	0.191	0.00 (0-0.004)	0.011	0.125 (0.003-0.255)	0.762
Baseline RNFL	0.011 (0.001-0.028)	0.121	0.024 (0.003-0.050)	0.642	0.009 (0.002-0.023)	0.381
Baseline macula	0.011 (0.002-0.026)	0.141	0.019 (0-0.043)	0.257	0.010 (0.002-0.023)	0.551
Baseline ODP + RNFL	0.023 (0.010-0.043)	0.996	0.021 (0.003-0.047)	0.359	0.010 (0.002-0.022)	0.271
Baseline ODP + macula	0.008 (0.001-0.025)	0.127	0.029 (0.007-0.054)	0.864	0.011 (0.003-0.025)	0.624
Baseline RNFL + macula	0.010 (0.002-0.029)	0.237	0.017 (0.00-0.041)	0.374	0.012 (0.003-0.025)	0.729
Baseline ODP + RNFL + macula	0.023 (0.010-0.042)	—	0.031 (0.008-0.058)	—	0.014 (0.005-0.026)	—

ODP = optic disc photo; RNFL = retinal nerve fiber layer; AUC = area under receiver characteristic curve
* P-values were from the comparison of each model with the model evaluating all three structural data.
