# Supplementary material for: An Artificial Intelligence-Based Prognostic Model for Prediction of Functional Glaucoma Progression From Clinical and Structural Data
Source: Am J Ophthalmol. Author manuscript; Available in PMC 2026 Jul 17. (PMC13379235; doi:10.1016/j.ajo.2025.12.026)
Supplement: 6 [file NIHMS2189849-supplement-6.docx]

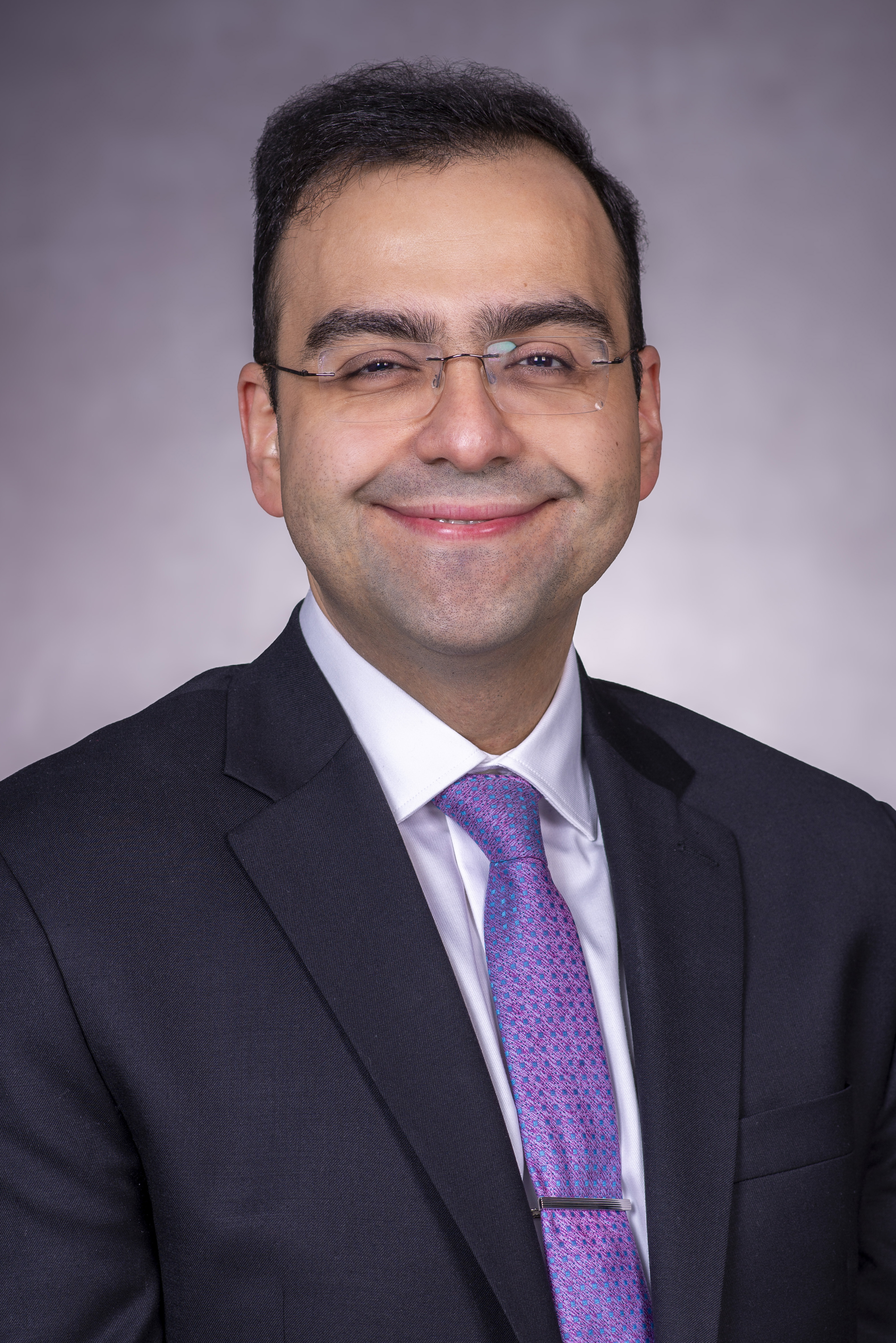


Vahid Mohammadzadeh, MD, is an ophthalmology resident at the University of Louisville. He completed medical training at Tehran University of Medical Sciences and finished his first ophthalmology residency at Farabi Eye Hospital. His academic interests include glaucoma diagnostics, AI-based prediction of glaucoma progression, and AI-enabled ophthalmic imaging. He foresees himself becoming a clinician-scientist who will contribute to high-quality patient care and innovation in glaucoma diagnosis and management.
